# PAK4 phosphorylating RUNX1 promotes ERα-positive breast cancer-induced osteolytic bone destruction

**DOI:** 10.7150/ijbs.47225

**Published:** 2020-05-25

**Authors:** Lina Tang, Yunling Gao, Yongqi Song, Yang Li, Yanshu Li, Hongyan Zhang, Danni Li, Jiabin Li, Caigang Liu, Feng Li

**Affiliations:** 1 Department of Cell Biology, Key Laboratory of Cell Biology of National Health Commission of the PRC, and Key Laboratory of Medical Cell Biology of Ministry of Education of the PRC, China Medical University, No.77, Puhe Road, Shenyang, 110122, Liaoning, China.; 2 Department of Breast Surgery, Shengjing Hospital of China Medical University, Shenyang, 110001, China.; 3 Department of Medical Oncology, The First Hospital of China Medical University, Shenyang, 110001, China.

**Keywords:** PAK4, RUNX1, phosphorylation, osteolytic bone destruction

## Abstract

The biological function of nuclear PAK4 in ERα-positive breast cancer osteolytic bone destruction remains unclear. Here, we find that the nuclear PAK4 promotes osteoclastogenesis and tumor-induced osteolysis via phosphorylating RUNX1. We show that nuclear PAK4 interacts with and phosphorylates RUNX1 at Thr-207, which induces its localization from the nucleus to the cytoplasm and influences direct interaction with SIN3A/HDAC1 and PRMT1. Furthermore, we reveal that RUNX1 phosphorylation by PAK4 at Thr-207 promotes osteolytic bone destruction via targeting downstream genes related to osteoclast differentiation and maturation. Importantly, we verify changes in RUNX1 subcellular localization when nuclear PAK4 is positive in breast cancer bone metastasis tissues. Functionally, we demonstrate that RUNX1 phosphorylation promotes osteolytic bone maturation and ERα-positive breast cancer-induced osteolytic bone damage in the mouse model of orthotopic breast cancer bone metastasis. Our results suggest PAK4 can be a therapeutic target for ERα-positive breast cancer osteolytic bone destruction.

## Introduction

Approximately 75% of breast cancers are ER positive and breast cancer patients most commonly develop bone metastases and forms osteolytic lesions 1,2. Significant progress has been made in breast cancer research in recent decades. While, the mechanism of cancer cell and bone microenvironment interaction and how to generate osteolytic bone lesions still need to be explored. As a serine/threonine protein kinase, P21-activated kinase 4 (PAK4) is considered to be one of the key regulators of the signaling network in tumor cells and participates in important cell life activities, such as cytoskeletal organization, cell motility, cell cycle regulation and transformation 3,4. Studies indicate that PAK4 is commonly highly expressed in a variety of cancers, including breast cancer. High PAK4 activity promotes tumor development 5-7. Studies have shown that PAK4 modulates breast cancer tumorigenesis by activating PI3K/AKT signaling 8. In addition, in breast cancer cells, PAK4 promotes migration and invasion by activating CEBPB 9. Likewise, nuclear PAK4 represses the expression of LIFR to promote the bone metastasis of ER positive breast cancer cells 10. Although this study has suggested that nuclear PAK4 modulates breast cancer bone metastasis, the underlying molecular mechanism for osteolytic bone destruction remains unclear.

Runt-related transcription factor 1 (RUNX1) has been found as a key regulator in hematopoietic malignancies 11-13. It has been shown that RUNX1 regulates EMT and inhibits tumor progression 14. Nevertheless, the relationship between PAK4 and RUNX1 in breast cancer is still unclear.

Here, we find that PAK4 phosphorylates RUNX1, which upregulates downstream genes related to osteoclast differentiation and maturation, including Jagged-1,IL-1α,IL-1β,IL-6 and PTHrP in breast cancer cells. The results demonstrate a novel mechanism underlying PAK4 promoting ERα-positive breast cancer-induced osteolysis in the bone microenvironment, suggesting that PAK4 may be a therapeutic target for ERα-positive breast cancer osteolytic bone destruction.

## Materials and methods

### Cell culture

Human breast cancer cell lines MCF-7 and ZR-75-30 were purchased from Shanghai cell bank of CAS-Chinese Academy of Sciences, and cells were cultivated in MEM or RPMI-1640 (Life, Shanghai, China). Mouse osteoblast cell line MC3T3-E1 and mouse macrophage cell line RAW264.7 were purchased from Beijing cell bank of CAS-Chinese Academy and cultured in MEM or DMEM. The mediums were supplemented with 10% FBS. For E2-free cell culture experiments, cells in (-E2) group were cultured in phenol red-free MEM supplemented with 5% dextran-charcoal-stripped fetal calf serum.

### Plasmid construction

GFP-RUNX1 construct was purchased from GeneChem Company (Shanghai, China). Flag-PAK4 (WT, NE, KM), Flag-RUNX1 (WT, T207A and T207D mutant), GST-PAK4, GST-RUNX1 constructs were inserted into pcDNA3.1-Flag and pGEX-5X-1 vectors.

### Lentiviral production and infection

Lentiviruses harboring PAK4, PAK4 shRNA-lentivirus, RUNX1 WT, RUNX1 T207A and RUNX1 T207D were constructed by GeneChem Company (Shanghai, China). Cells were infected with lentiviral and 1 μg/ml puromycin (Sigma, St Louis, USA) were used to select stable cells.

### Antibodies

PAK4 (Cell Signaling Technology, USA); PAK4 (Santa cruz); Phospho-PAK4 (Ser474,Cell Signaling Technology, USA); RUNX1 (Abcam, USA), RUNX1 (Santa cruz), SIN3A (Cell Signaling Technology, USA); HDAC1 (Cell Signaling Technology, USA); PRMT1 (Cell Signaling Technology, USA); Mono-Methyl Arginine (R*GG) (Cell Signaling Technology, USA); Jagged-1 (Cell Signaling Technology, USA); PTHrP (Abcam, USA); Flag, GFP, GAPDH (GenScript, Nanjing, China).

### PAK4 kinase assay

GST-fusion proteins were purified in vitro and washed with kinase buffer (50 mM HEPES, pH 7.5, 10 mM MgCl2, 2 mM MnCl2 and 0.2 mM DTT). The PAK4 kinase domain was synthesized in cells and used in the kinase assay; 50 μl of kinase buffer, 10 μCi of [γ-32P] ATP (5000 Ci/mmol) and 2.5 μM cold ATP were also used for the reaction for 30 min at 30°C. After all proteins were separated on 12% SDS-PAGE gels and transferred onto PVDF membranes, ^32^P-labeled proteins were visualized via autoradiography. Myelin basic protein (MBP) was used as a positive control. The GST-fusion proteins were stained with Ponceau S.

### Immunoprecipitation, western blot analysis and GST pull-down assays

For immunoprecipitation, total protein lysate (2 mg) was used for each immunoprecipitation using specific antibody, Protein A agarose beads (GE Healthcare Uppsala, Sweden) were added to the cells and incubated overnight 4°C. Washed precipitated proteins were analyzed by western blot. The immunoprecipitation (IP), western blot and GST pull-down assays used in this study have been described previously in detail 15. A Glutathione-conjugated Sepharose bead (Amersham Biosciences, USA) was used to purificates the GST and GST-fusion proteins.

### Immunofluorescence analysis

Cells were fixed in methanol at room temperature for 20 min and then blocked with normal goat serum for 30 min. Cells were stained with PAK4, RUNX1 or Flag antibodies overnight at 4°C, Alexa Fluor 488 and 546 (Invitrogen, USA) and the DNA dye DAPI were used as secondary antibodies.

### RNA-seq analysis and Real-time PCR

TRIzol reagent (TaKaRa, Japan) was used to extracts the total RNA from breast cancer cells. RNA-seq analysis was performed at Gene Denovo (Guangzhou, China).

Samples after reverse transcription were amplified with specific primers in SYBR Green Mix (TaKaRa, Japan).

### Chromatin immunoprecipitation (CHIP) assays

Transfected cells were washed and cross-linked with 1% formaldehyde. The cells were sonicated to shear DNA and immunoprecipitated with anti-RUNX1 (Abcam, USA) antibodies and control immunoglobulin G at 4 °C overnight. After elution and reverse cross-linking, the purified DNA was resuspended in TE buffer. DNA samples (2 ul) were then amplified by PCR. This experiment was performed in MCF-7cells stably overexpressed PAK4 and the enrichment of target gene promoter pulled down by RUNX1 antibody.

### Immunohistochemistry

90 cases of BMBC specimens were obtained from the Department of Breast Surgery in the Shengjing Hospital of China Medical University. Immunohistochemistry assay has been described previously in detail 15.

### Chemotactic migration assay

Transwell inserts (Falcon, 8.0-µm pore size) in 24-well plate were used to perform the assay. MC3T3-E1 cells were cultured in the lower chamber and stable cancer cells in the upper chamber which migrated for 24 h. Count migrated cells under a microscope using a 20× objective. The experiment was repeated three times independently.

### Heterogeneous cell-cell adhesion assay

When the pre-seeded MC3T3-E1 cells density reached nearly 100% in a 24-well plate, the cancer cells transfected with GFP-tag plasmid were seeded on the MC3T3-E1 cells and cultured for 30-40 min and then remove the suspended cancer cells. The adherent cells were counted under a microscope using a 20× objective.

### Osteoclastogenesis assay and TRAP stain

RAW264.7 cells were cultured with 50ng/ml RANKL (R&D Systems, Minneapolis, MN) in a 12-well plate for 2 days. Then change the medium to cancer cells conditioned medium for another 6 days. TRAP stain (Sigma, St. Louis, MO) was used to identify multinuclear cells. The number of TRAP+ mature osteoclasts cells (≥3 nuclei) were counted under a microscope using a 20× objective and were analyzed using ImageJ.

### Mouse model

Female six to eight-week-old BALB/c nude mice (8 per group) were purchased from Vital River Laboratories (Beijing, China) were randomly split into four groups. The right tibiae of mice in both groups were injected with MCF-7 cells (5×10^6^) under appropriate anaesthetics (Tribromoethanol). E2V (20ug per dose, 0.1 ug/ul in H2O) were administered by oral gavage every day. All mice were sacrificed on day 45 (carbon dioxide euthanasis), tibiae tissues were collected for X-ray and TRAP analysis. All experimental procedures involving animals were conducted in accordance with the Guide for the Care and Use of Laboratory Animals (NIH publication no. 80-23, revised 1996) and were performed according to the institutional ethical guidelines for animal experiments.

### Ser/Thr phosphoprotein purification assay

This assay used PhosphoProtein Purification Kit (Qiagen No. 37101) with a volume of lysate containing 2.5 mg of total protein. Finally, 30ul, 0.1 mg/ml concentrating protein was used for western blot. MCF-7 cells transfected with Flag-RUNX1 WT, Flag-RUNX1 T207A and GFP-PAK4 WT.

### Statistical analysis

Statistical analyses were performed using GraphPad Prism software. In repeated studies, student's t-test was used to analyze differences between two groups. *P<0.05 was considered statistically significant, and **P<0.01 and ***P<0.001 were considered highly significant.

## Results

### RUNX1 is phosphorylated by PAK4 at T-207

**Figure 1:**
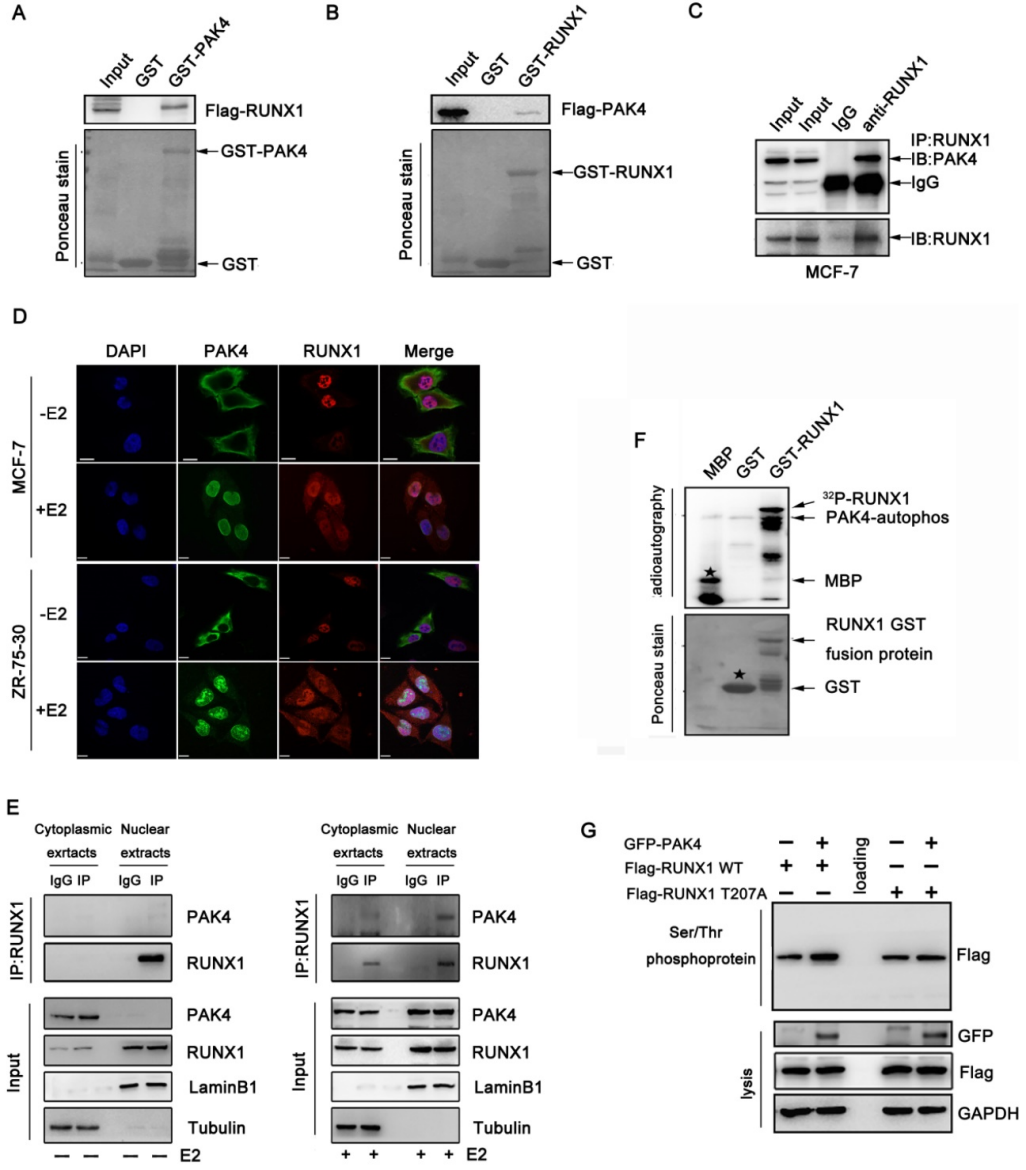
** PAK4 phosphorylates RUNX1 at T207.** (**A-B**) Recombinant human RUNX1 (A) or PAK4 (B) was incubated with bacterially expressed GST-PAK4 (A) or GST-RUNX1 (B). Western blotting was performed to evaluate the interaction. (**C**) Endogenous PAK4 and RUNX1 were evaluated in MCF-7 cells. Cell lysates were immunoprecipitated with RUNX1 antibodies or IgG. Precipitates were analyzed by western blot using the indicated antibodies. (**D**) Representative PAK4 and RUNX1 immunostaining in MCF-7 (upper) and ZR-75-30 (lower) cells cultured with or without E2. PAK4 (Alexa Flour 488 green); RUNX1 (Alexa Flour 546 red); and nuclei were stained with DAPI (blue). Merged images are shown as indicated. (**E**) Co-IP of PAK4 and RUNX1 from the nuclear and cytoplasmic fractions obtained from human MCF-7 cells cultured with or without E2. β-tubulin and LaminB1 were used as controls for the cytoplasmic and nuclear compartments, respectively. (**F**) An in vitro kinase assay using purified MBP, GST, and GST-RUNX1 fusion proteins as substrates for commercially available PAK4 kinase was performed. MBP served as a positive control. Phosphorylation was detected with autoradiography.The star symbol in the upper picture represents MBP, and the star symbol in the lower picture represents GST. (**G**) MCF-7 cells transfected with Flag-RUNX1 WT, Flag-RUNX1 T207A and GFP-PAK4 WT were used for Ser/Thr phosphoprotein purification. Then concentrated protein was used for western blot using the indicated antibodies,

Our previous study showed that several target genes of RUNX1, such as LIFR, E-cadherin, and β-catenin, are also regulated by PAK4^10^,^14^-^16^, suggesting that PAK4 may regulates the transcriptional activity of RUNX1. To confirm this hypothesis, the GST pull-down assay demonstrated the directly interaction between PAK4 and RUNX1 *in vitro* (Figure 1A and 1B). The coimmunoprecipitation studies in MCF-7 (Figure 1C) and ZR-75-30 cells (Supplementary Figure 1A) demonstrated the association of PAK4 with RUNX1. In our previous studies, we found that PAK4 translocated from the cytoplasm to the nucleus in the presence of 17β-estradiol (E2); the immunofluorescence studies indicated that there was no colocalization between PAK4 and RUNX1 in the absence of E2, but there was colocalization between PAK4 and RUNX1 in the nucleus in MCF-7 (Figure 1D upper) and ZR-75-30 (Figure 1D lower) cells in the presence of E2. Furthermore, the cell fractionation studies indicated that PAK4 was associated with RUNX1 in the nucleus compartment of MCF-7 cells in physiological conditions (Figure 1E, right), whereas, no physical interaction between PAK4 and RUNX1 was detected without E2 (Figure 1E, left). For estrogen treatment experiments, cells in +E2 group were first cultured in phenol red-free MEM supplemented with 5% dextran-charcoal-stripped fetal calf serum for 48h, and then cells were cultured in MEM supplemented with 10% FBS. In all subsequent cell experiments, if there is no explicit labeling of estrogen-free, follow this experimental method. Well known that PAK4 is a serine/threonine protein kinase, so we want to determine whether PAK4 phosphorylated RUNX1. According to the GPS software forecast and bioinformatics analysis, Thr-207 is the highest-rated phosphorylation site and has important biological significance, such as cell localization and transcriptional regulation of RUNX1, so we chose Thr-207 as the main phosphorylation site for further research and we created a single-site mutation RUNX1 T207A. The *in vitro* kinase assays was used to confirm that PAK4 can phosphorylate RUNX1 (Figure 1F). Then, PAK4-mediated RUNX1 phosphorylation was further tested in whole cell by Serine/Threonine phosphoprotein purification kit (Figure 1G). According to the GPS software forecast, we created a single-site mutation RUNX1 T207A. The western blot results showed that phosphorylation level of wild type RUNX1 but not RUNX1 mutant T207A was increased with overexpression of PAK4 (Figure 1G, the top lane, compare lane 2 with lane 1 and compare lane 5 with lane 4). These results indicate that PAK4 interacts with RUNX1 and phosphorylates it at T207 in the nucleus in physiological conditions.

### Phosphorylation of RUNX1 at T-207 induces its translocation from the nucleus to the cytoplasm

**Figure 2:**
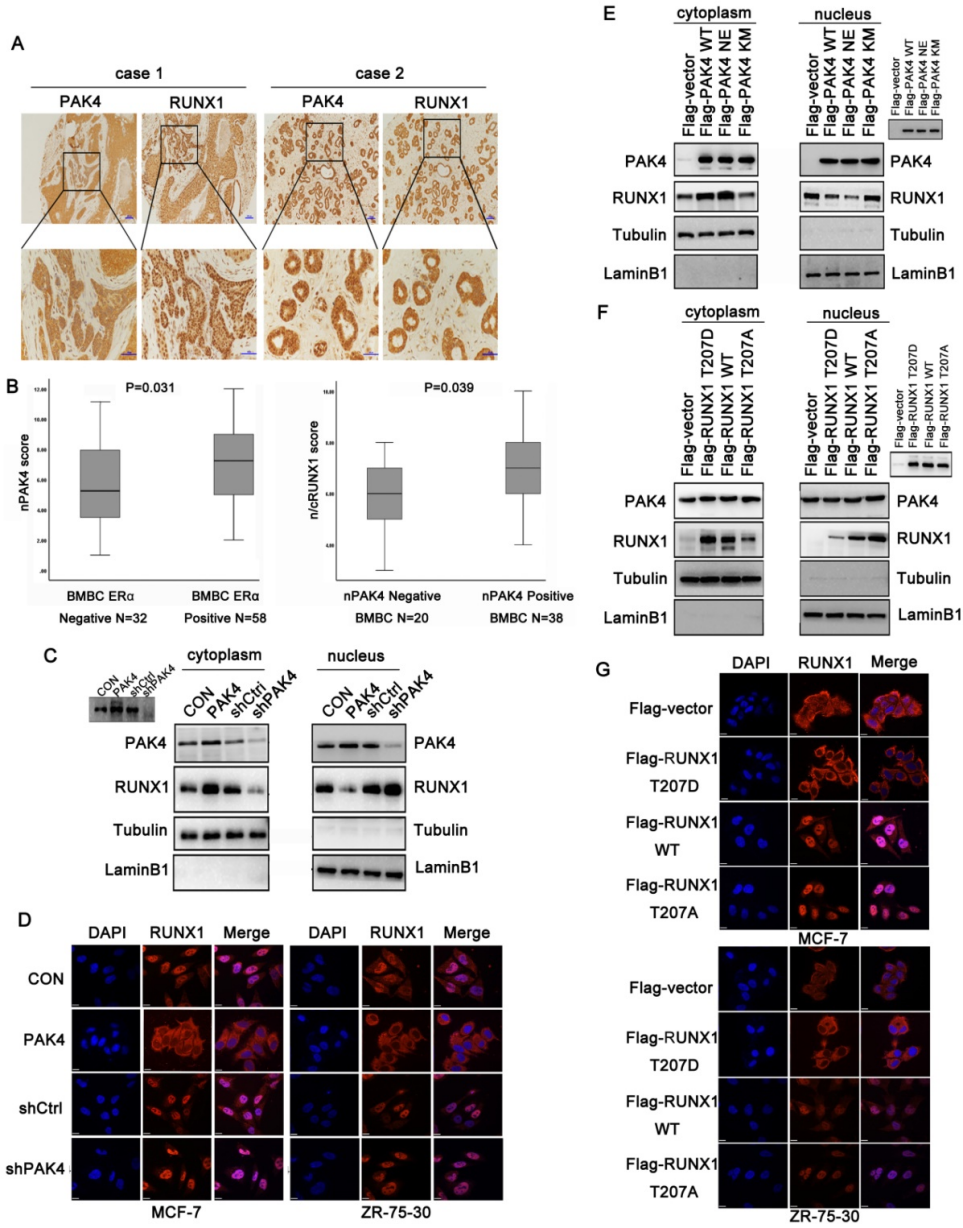
** Phosphorylation of RUNX1 at T207 induces its translocation from the nucleus to the cytoplasm.** (**A**) Representative images showing negative nuclear PAK4 with positive nuclear RUNX1 (case1) or positive nuclear PAK4 with nuclear and cytoplasmic RUNX1 (case2) localization. Scale bars, 50 µm. (**B**) Box plot of nPAK4 in BMBC samples from 90 subjects. The subjects were divided into two groups based on ERα expression (left). Box plot of nuclear and cytoplasmic RUNX1 in 58 ER positive BMBC samples. The subjects were divided into two groups based on nuclear PAK4 expression (right). The data were analyzed using the Mann-Whitney U test. The horizontal lines represent the median; the bottom and top of the boxes represent the 25th and 75th percentiles, respectively, and the vertical bars represent the range of the data. (**C**) Western blot of RUNX1 from the nuclear and cytoplasmic fractions obtained from human MCF-7 cells stably overexpression or knockdown of PAK4. β-tubulin and LaminB1 were used as controls for the cytoplasmic and nuclear compartments, respectively. Insets represent western blot analyses of PAK4. (**D**) MCF-7 (left) and ZR-75-30 (right) cells stably overexpression or knockdown of PAK4, RUNX1 (Alexa Flour 546 red); and nuclei were stained with DAPI (blue). Merged images are shown as indicated. (**E**) Western blot of RUNX1 from the nuclear and cytoplasmic fractions obtained from human MCF-7 cells transfected with Flag-PAK4 WT, Flag-PAK4 NE, or Flag-PAK4 KM, Insets represent western blot analyses of the exogenous FLAG-tagged proteins. (**F**) Western blot of RUNX1 from the nuclear and cytoplasmic fractions obtained from human MCF-7 cells transfected with Flag-RUNX1 T207D, Flag-RUNX1 WT or Flag-RUNX1 T207A Insets represent western blot analyses of the exogenous FLAG-tagged proteins. (**G**) MCF-7 (upper) and ZR-75-30 (lower) cells transfected with Flag-RUNX1 T207D, Flag-RUNX1 WT or Flag-RUNX1 T207A, Flag (Alexa Flour 546, red), and nuclei were stained with DAPI (blue). Merged images are shown as indicated.

A previous study showed that RUNX1 localized in the nuclear matrix 17. According to the immunofluorescence (Figure 1D) and cell fractionation studies (Figure 1E right) shown above, when PAK4 entered the nucleus with E2 stimulation, the cytoplasmic localization of RUNX1 increased. While RUNX1 expression has not increased with E2 treatment (Supplementary Figure 1E). Therefore, we hypothesized that PAK4 entry into the nucleus influences RUNX1 subcellular localization. Our previous studies have shown that nuclear PAK4 promotes ERα positive breast cancer bone metastasis 10. Therefore, we assumed that the changes in subcellular localization of PAK4 and RUNX1 may be associated with bone metastases. To verify the significance of subcellular localization of PAK4 and RUNX1 in bone metastatic breast cancer (BMBC), immunohistochemical staining in specimens from 90 cases of BMBC were used to evaluate the subcellular localization of PAK4 and RUNX1; IHC staining studies showed that when nuclear PAK4 was negative, RUNX1 was mainly located in the nucleus (Figure 2A, case 1), while positive nuclear expression of PAK4 induced RUNX1 translocation from the nucleus to the cytoplasm (Figure 2A, case 2). Nuclear PAK4 positivity was greater in ERα positive BMBCs than in ERα- negative BMBCs (Fig. 2B left; P = 0.031). The positivity of RUNX1 located in both the nucleus and cytoplasm (n/cRUNX1) was greater in nuclear PAK4-positive BMBCs than that in nuclear PAK4-negative BMBCs (Fig. 2B right; P = 0.039). The same phenomenon was also detected in the nucleocytoplasmic separation western blot studies in both MCF-7 (Figure 2C) and ZR-75-30 cells (Supplementary Figure 1B), and the immunofluorescence studies in both MCF-7 (Figure 2D left) and ZR-75-30 (Figure 2D right) cells that stably overexpressed or knocked down PAK4. According to the bioinformatics prediction, the R206 amino acid site in RUNX1 is a nuclear export signal (NES). Therefore, we hypothesized that the phosphorylation of RUNX1 at T207 influenced its subcellular localization. To analyze whether the translocation of RUNX1 depended on phosphorylation by PAK4, wild-type PAK4 (PAK4 WT), kinase active-type PAK4 (PAK4 NE) or kinase dead- type PAK4 (PAK4 KM) were transfected in MCF-7 and ZR-75-30 cells, the nucleocytoplasmic separation studies showed that PAK4 NE induced more RUNX1 translocation from the nucleus to the cytoplasmic than PAK4 KM (Figure 2E and Supplementary Figure 1C). To determine whether T207 is the main site phosphorylated by PAK4 that induced the translocation of RUNX1, MCF-7 and ZR-75-30 cells were transfected with wild-type RUNX1 (RUNX1 WT), phosphorylation-active mimic mutant RUNX1 (RUNX1 T207D) and phosphorylation-disabled mutant RUNX1 (RUNX1 T207A); the nucleocytoplasmic separation studies in MCF-7 (Figure 2F) and ZR-75-30 cells (Supplementary Figure 1D) , and the immunofluorescence studies in both MCF-7 (Figure 2G upper) and ZR-75-30 (Figure 2G lower)cells showed that RUNX1 T207D translocated from the nucleus to the cytoplasm more readily than RUNX1 WT, while RUNX1 T207A was mainly located in the nucleus. These results suggest that RUNX1 phosphorylation at T207 by PAK4 influences its subcellular localization.

### Phosphorylation of RUNX1 at T207 influences interaction with both SIN3A/HDAC1 and PRMT1

**Figure 3:**
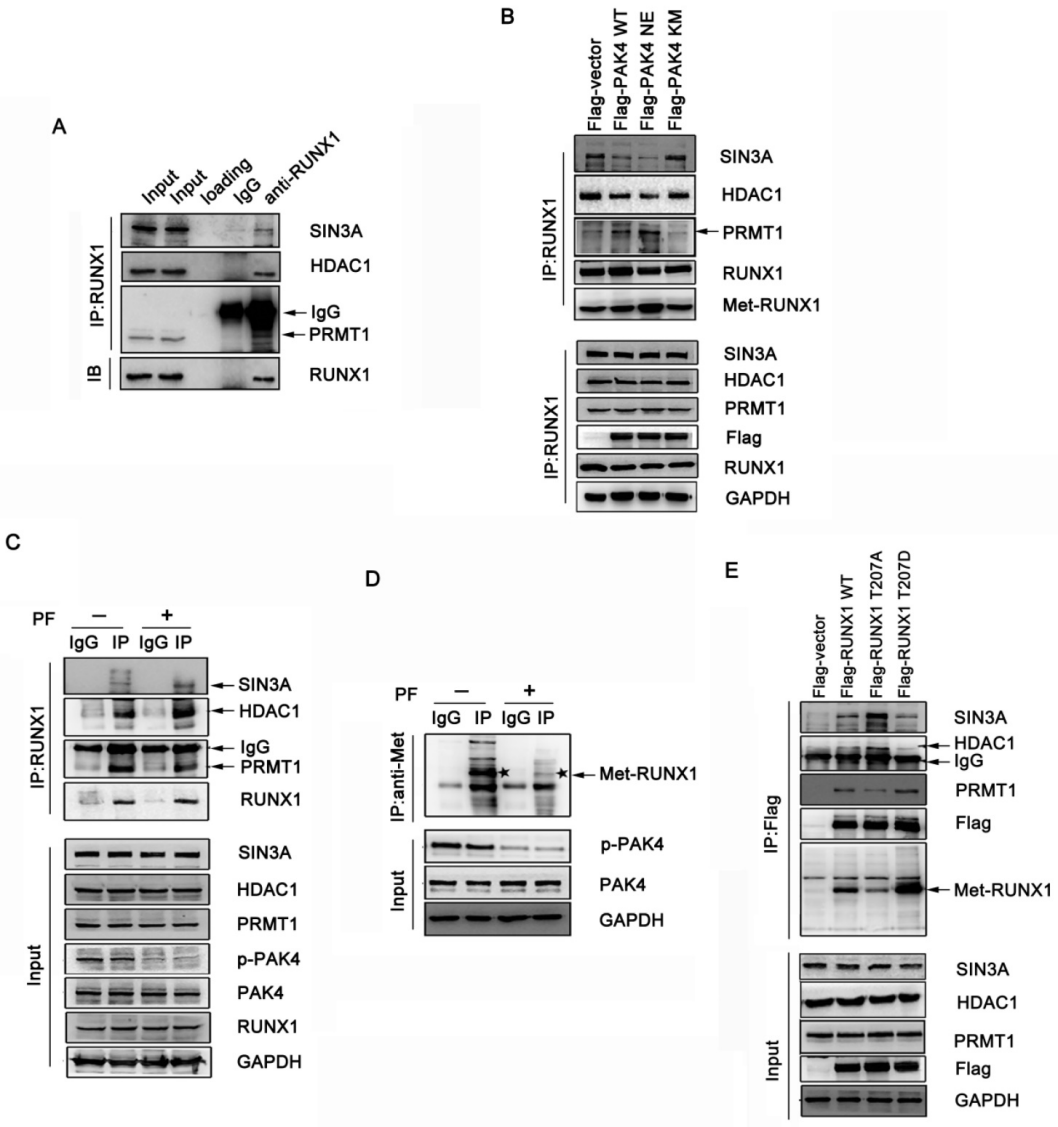
** Phosphorylation of RUNX1 at T207 influences interaction with both SIN3A/HDAC1 and PRMT1.** (**A**) MCF-7 cell lysates were immunoprecipitated with RUNX1 antibodies or IgG. Precipitates were analyzed by western blot using the indicated antibodies. (**B**) MCF-7 cells transfected with Flag-PAK4 WT, Flag-PAK4 NE, or Flag-PAK4 KM were immunoprecipitated with the RUNX1 antibodies or IgG. Precipitates were analyzed by western blot using the indicated antibodies. (**C-D**) MCF-7 cells treat with PF-3758309 (5Μm, 14h), cell lysates were immunoprecipitated with the RUNX1 antibodies or IgG (C) or with the methylation antibody or IgG and the star symbol in the picture represents Met-RUNX1 (D) . Precipitates were analyzed by western blot using the indicated antibodies. (**E**) MCF-7 cells transfected with Flag-RUNX1 WT, Flag-RUNX1 T207A or Flag-RUNX1 T207D were immunoprecipitated with the Flag antibodies. Precipitates were analyzed by western blot using the indicated antibodies.

It has been shown that RUNX1 associates with the Sin3A and HDAC1 corepressor complex, which represses target genes expression, and that PRMT1 associates with RUNX1 and methylates it at R206 and R210, which abrogates SIN3A/HDAC1 corepressor complex binding and promotes its transcriptional activity 18. Therefore, we hypothesized that the phosphorylation of RUNX1 at T207 may also influence its association with the SIN3A/HDAC1 corepressor complex. The coimmunoprecipitation studies demonstrated that endogenous RUNX1 was associated with endogenous SIN3A, HDAC1 and PRMT1 in MCF-7 cells (Figure 3A). To investigate whether the phosphorylation of RUNX1 by PAK4 affect the interaction described above, we performed immunoprecipitation studies in MCF-7 and ZR-75-30 cells that overexpressed PAK4-WT, PAK4-NE or PAK4-KM. We found that PAK4 NE more obviously repressed the interaction between RUNX1 and both SIN3A and HDAC1 than did PAK4 KM, and there was a positive relationship between the methylation and phosphorylation levels of RUNX1 (Figure 3B, Supplementary Figure 2A and 2B). Furthermore, the PF-3758309 (a PAK inhibitor) was used to inhibit PAK4 kinase activity, and the immunoprecipitation studies further demonstrated the inhibition of the interaction between RUNX1 and both SIN3A and HDCA1 corepressor complex and the enhancement of not only the association between RUNX1 and PRMT1 but also the methylation of RUNX1, which was dependent of the PAK4 kinase activity (Figure 3C and 3D). To further define whether T207 is the main site that induced the affect above, MCF-7 and ZR-75-30 cells were transfected with RUNX1 WT, RUNX1 T207A or RUNX1 T207D, and we found that RUNX1 T207D upregulated the association with PRMT1 and that its methylation level was significantly upregulated. RUNX1 T207A had the opposite effect (Figure 3E, Supplementary Figure 2C). These findings indicate that PAK4-mediated RUNX1 phosphorylation at T207 promotes the RUNX1-PRMT1 interaction and upregulates RUNX1 methylation level.

### RUNX1 phosphorylation at T207 regulates the expression of genes related to osteoclast differentiation and maturation

**Figure 4:**
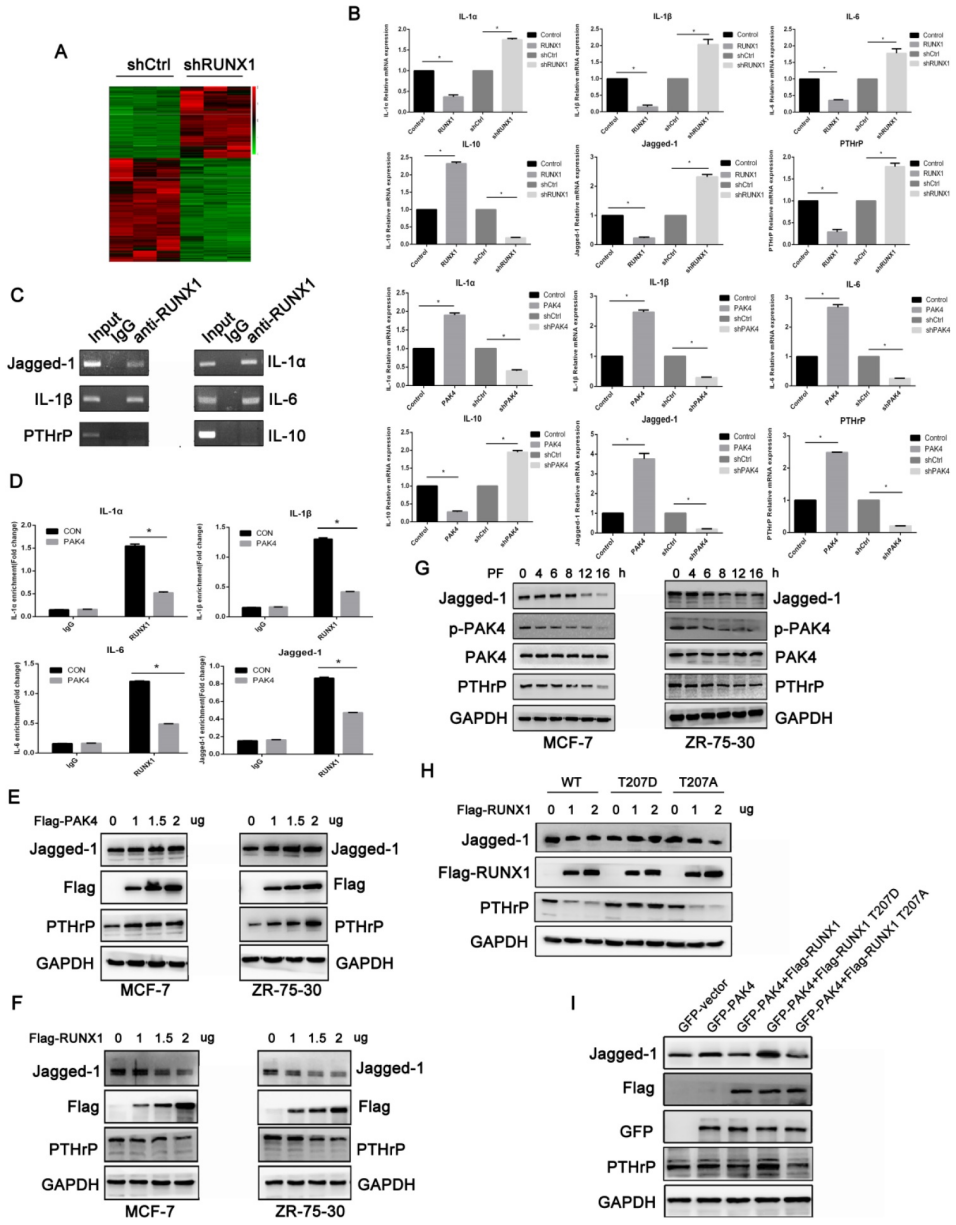
** RUNX1 phosphorylation at T207 regulates the expression of genes related to osteoclast differentiation and maturation.** (**A**) Heat map displays the altered expression of genes upon RUNX1 knockdown in MCF-7 cells. Fold change is indicated at right. (**B**) Real-time PCR (RT-PCR) analysis of MCF-7 cells stably overexpressing or knocked down RUNX1 or PAK4 to vivificated the six target genes of RNA-seq. (**C**) In the ChIP assay, primary antibodies against IgG and RUNX1 were used to immunoprecipitate the DNA sequences in the lysate of MCF-7 cells, and specific primers were used to detect the target gene promoter sequence. (**D**) MCF-7 cells stably overexpressing PAK4. ChIP assays were performed to determine the binding of RUNX1 in the promoter of target genes. The enrichment of target gene promoter pulled down by RUNX1 antibody was analyzed by qRT-PCR and normalized to the value of control vector or RUNX1 group. Data are shown as mean ± SD. *P < 0.05, **P < 0.01. (**E**) MCF-7 (left) and ZR-75-30 (right) cells were transiently transfected with control or increasing amounts of Flag-PAK4 expression plasmid. Jagged-1, PTHrP and PAK4 were detected by western blotting. GAPDH was used as a loading control. (**F**) MCF-7 (left) and ZR-75-30 (right) cells were transiently transfected with control or increasing amounts of Flag-RUNX1 expression plasmid. (**G**) MCF-7 (left) and ZR-75-30 (right) cells treated with PF-3758309 (5Mm) were subjected to the western blotting assay with indicated antibodies. (**H**) MCF-7 cells transiently transfected with control or increasing amounts of Flag-RUNX1 WT, Flag-RUNX1 T207D or Flag-RUNX1 T207A were subjected to the western blotting assay with indicated antibodies. (**I**) MCF-7 cells transfected with GFP-PAK4, both GFP-PAK4 and Flag-RUNX1 WT, both GFP-PAK4 and Flag-RUNX1 T207D or both GFP-PAK4 and Flag-RUNX1 T207A were subjected to the western blotting assay with indicated antibodies.

Our results demonstrated that PAK4 abrogated RUNX1-mediated transcription repression by phosphorylating T207. To explore the genes related to osteoclast differentiation and maturation of the PAK4-RUNX1 axis. RNA sequencing (RNA-seq) with differentially expressed genes analysis was used to detect target genes of RUNX1 (Figure 4A). According to the gene expressing data, we selected six genes that have been reported to be related to osteoclast differentiation and maturation, including Jagged-1, IL-1α, IL-1β, IL-6 , IL-10 and PTHrP. Using quantitative PCR (qPCR) to verify the RNA-seq results, we found that overexpression of RUNX1 downregulated these genes that induced osteoclast differentiation, such as Jagged-1,IL-1α,IL-1β,IL-6 and PTHrP, but upregulated IL-10 which has anti-osteoclastogenic effects. Then, we overexpressed PAK4 to identify its effect, and the qPCR results showed that PAK4 had an opposite effect compared with RUNX1 (Figure 4B).The interactions between RUNX1 and these target genes were confirmed by CHIP assays (Figure 4C and 4D) .Finally, we focused on Jagged-1, which stimulated osteoclastogenesis and bone degradation^19^. Western blot analysis showed that the addition of Flag-PAK4 dose-dependently upregulated Jagged-1 and PTHrP expression in both MCF-7(Figure 4E left) and ZR-75-30 cells (Figure 4E right). RUNX1 dose-dependently downregulated Jagged-1 and PTHrP expressions in both MCF-7 (Figure 4F left) and ZR-75-30 cells (Figure 4F right). Moreover, we examined whether upregulation of Jagged-1 and PTHrP requires PAK4 kinase activity. MCF-7 and ZR-75-30 cells were treated with PF-3758309, and the results indicated that the regulation of Jagged-1 and PTHrP by PAK4 depended on its kinase activity (Figure 4G). To further define whether upregulation of Jagged-1 and PTHrP requires PAK4-mediated RUNX1 phosphorylation, increasing doses of RUNX1 WT, RUNX1 T207D or RUNX1 T207A were transfected into MCF-7 cells, and the results indicated that RUNX1-driven Jagged-1 and PTHrP downregulation were abrogated by RUNX1 phosphorylation at T207(Figure 4H). Finally, we found that the upregulation of Jagged-1 and PTHrP by PAK4 could be reversed by RUNX1 WT and RUNX1 T207A instead of RUNX1 T207D, and RUNX1 T207A had a more obvious effect than RUNX1 WT (Figure 4I). In summary, these results suggest that the phosphorylation of RUNX1 by PAK4 up-regulates the expression of genes which relate to osteoclast differentiation and maturation.

### Phosphorylation of RUNX1 at T207 influences the interaction of breast cancer cells with the bone microenvironment *in vitro*

**Figure 5:**
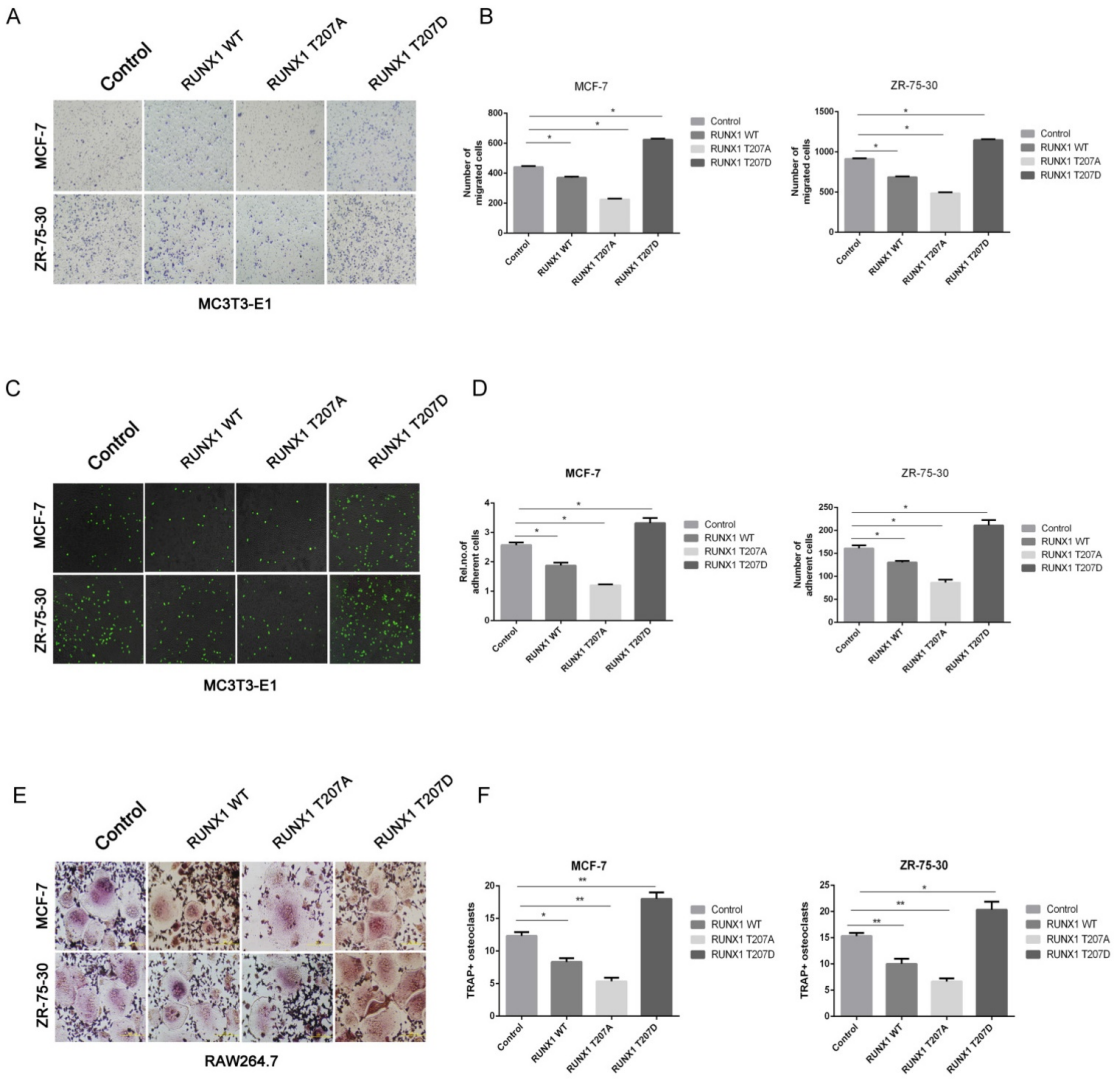
** Phosphorylation of RUNX1 at T207 influences the interaction of breast cancer cells with the bone microenvironment *in vitro.* MCF-7 and ZR-75-30 cells stably overexpressed control, RUNX1 WT, RUNX1 T207A or RUNX1 T207D.** (**A-B**) Chemotactic migration of MCF-7 (upper) and ZR-75-30 (lower) cancer cells toward MC3T3E1 cells was assessed by transwell assays (A) Results are representative of three independent experiments. *p < 0.05, **p < 0.01 (B). (**C-D**) Adhesion of MCF-7 (upper) and ZR-75-30 (lower) cancer cells to MC3T3-E1 cells was assessed by adding cancer cells to MC3T3E1 cells at 100% saturation, followed by incubation for 30-40 min (C). Results are representative of three independent experiments. *p < 0.05, **p < 0.01 (D). (**E-F**) The ability of cancer cells to induce osteoclast differentiation was assessed by incubation of RAW264.7 cells with MCF-7 (upper) and ZR-75-30 (lower) cancer cell CM (E). The number of mature osteoclasts with TRAP positive multinucleated (≥3 nuclei) were analyzed. Results are representative of three independent experiments. *p < 0.05, **p < 0.01 (F).

To explore the role of RUNX phosphorylation in the interaction of breast cancer cells with the bone microenvironment, we evaluated various features of cancer cells. MC3T3-E1 and breast cancer cells were used for the chemotactic migration experiments, the results showed that MCF-7 and ZR-75-30 cells overexpressing RUNX1 WT repressed migration of cancer cells towards MC3T3-E1 cells compared with control, and the overexpression of RUNX1 T207A showed a marked suppressive effect compared with RUNX1 WT, while the overexpression of RUNX1 T207D significantly promoted cancer cells migrate to MC3T3E1 cells (Figure 5A and 5B). Furthermore, overexpression of RUNX1 T207A showed an obvious repressive effect on heterogeneous cell-cell adhesion, while overexpression of RUNX1 T207D showed an opposite effect (Figure 5C and 5D). Because breast cancer cells bone metastasis commonly generates osteolytic lesions, we tested whether phosphorylation of RUNX1 at T207 regulates cancer-induced osteoclastogenesis. RAW264.7 cells were incubated with CM from stably transfected cells cancer. TRAP staining showed that the RUNX1 T207D-overexpressing cancer cells-induced TRAP^+^ osteoclasts number was significantly increased compared with other cells (Figure 5E and 5F). Together, these results indicate that RUNX1 phosphorylation at T207 modulates ER positive breast cancer cells bone-specific metastatic potential.

### Phosphorylation of RUNX1 at T207 promotes osteolytic bone destruction *in vivo*

**Figure 6:**
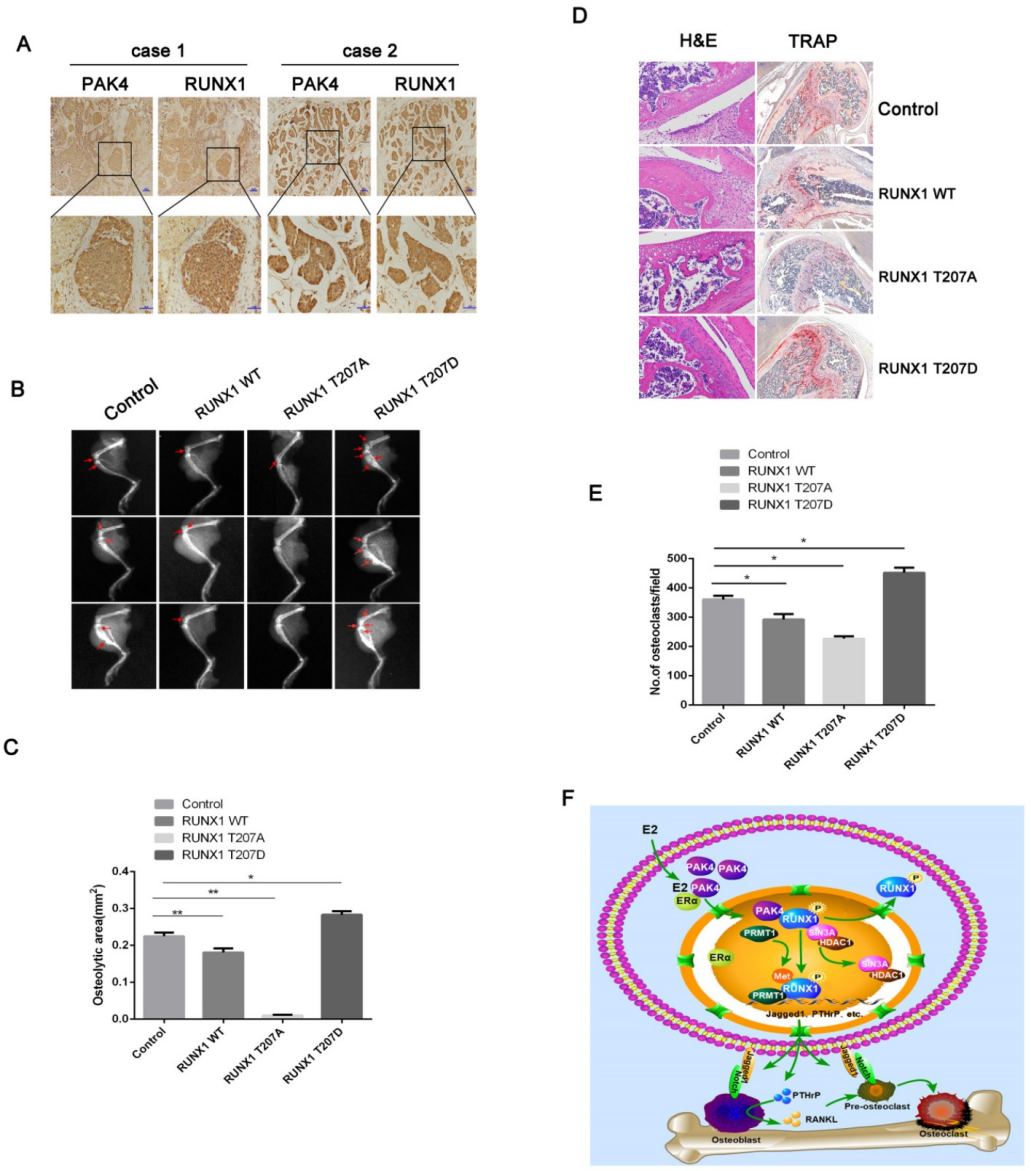
** Phosphorylation of RUNX1 at T207 promotes breast cancer bone destruction *in vivo.***(**A**) Representative Immunohistochemistry staining with PAK4 and RUNX1 in bone metastatic tissues. Scale bars, 50 µm. (**B-E**) X-ray imaging of mice injected with MCF-7cells treated as indicated were analyzed. Arrows point to osteolytic lesions (B). *P < 0.05 by Student's t test. Error bars are defined as s.d (C). The metastases and osteoclasts in the hind leg bones of mice injected with MCF-7 cells treated as indicated were visualized by H&E and TRAP staining, respectively (D) Scale bars 50 µm for H&E and 200 µm for TRAP. *P < 0.05 by Student's t test. Error bars are defined as s.d (E). (**F**) Schematic representation of PAK4 and RUNX1 functions in osteoclastogenesis and tumor-induced osteolysis in ERα-positive breast cancer bone destruction.

Two cases of bone metastatic tissue stained by IHC showed the location of positive nuclear PAK4 and n/cRUNX1 (Figure 6A).To investigate how PAK4 modulates the osteolytic destruction through RUNX1 *in vivo*, we used the breast cancer cell line MCF-7 to establish a tumor-induced osteolysis model, which stably overexpressed RUNX1 WT, RUNX1 T207A or RUNX1 T207D. As shown by X-ray photography (Figure 6B and 6C) and verified by H&E and TRAP staining (Figure 6D and 6E), breast cancer cells induced osteolytic bone destruction, and RUNX1 T207D overexpression significantly enhanced osteolytic bone destruction compared with control, whereas RUNX1 T207A overexpression markedly reversed the osteolytic bone destruction in limbs. These results indicate that the phosphorylation of RUNX1 by PAK4 at T207 by PAK4 promotes breast cancer osteolytic bone destruction.

## Discussion

This study demonstrates that in ERα-positive breast cancer, nuclear PAK4 interacts with and phosphorylates RUNX1 in the nucleus; on the one hand, RUNX1 NES exposure changes the RNUX1 nucleoplasm distribution, and on the other hand, RUNX1 phosphorylation at T207 upregulates RUNX1 methylation by recruiting PRMT1 and further preventing the combination of RUNX1 with the corepressor complex SIN3A and HDAC1. The two aspects work together to relieve the transcriptional inhibition of RUNX1 on the target genes including IL-1α, IL-1β, IL-6, Jagged-1 and PTHrP, affecting the mature differentiation of osteoclasts and promoting osteolytic bone destruction. In summary, these findings demonstrate the molecular mechanism of PAK4 promoted osteolytic bone destruction in ERα positive breast cancer.

The interactions of tumor cells with bone microenvironment mediate osteolytic metastases and involve aberrant bone resorption 20. Breast cancers disrupt the balance between osteogenesis and osteolysis by releasing factors such as PTHrP, IL-6, MMPs that leading to bone destruction 21. Activated osteoblasts also release cytokines that activating osteoclast differentiation 22. Tumor-expressed Jagged-1 promotes osteoclast differentiation, and tumor-released cytokines amplifying the osteoclast differentiation and activity 23-27 .Bone destruction then secrets growth factors to promote tumor growth in bone 28,29. Therefore, finding the molecular mechanism and molecular target for regulating the interaction of breast cancer cells with bone microenvironment is a key point in treating of breast cancer bone metastasis.

Primary breast cancers with high Jagged-1 expression tend to develop bone metastasis 19,30. In human breast cancer, increased expression of Jagged-1 and Notch-1 are significantly correlated with poor prognosis 31. It has been reported that tumor-derived Jagged-1 activates Notch signaling and promotes bone metastasis, and upregulates IL-6 and TGF, which in turn promote tumor cells proliferation and survival 22,32,33.

As a member of RUNX gene family, RUNX1 is an important regulator for normal physiological functions. Loss of RUNX1 is linked to many types of cancers 34. For example, in ovarian and skin cancers, RUNX1 has been identified as an oncogene, while it exerted tumor suppressive effect in lung and prostate cancers 35-38. Notably, in renal carcinoma endometrial cancer, RUNX1 was reported to be associated with EMT 39,40. RUNX1 interacts with FOXO to regulate breast tissue morphogenesis and progression 41-44. It has been reported that RUNX1 suppresses breast cancer progression in ER-positive cells 45. And RUNX1 can be phosphorylated by Src, cdks and c-Abl 46-48. To date, the effect of RUNX1 phosphorylation on tumorigenesis and the clinical treatment of RUNX1 on breast cancer bone metastasis are uncertain. Our study confirms that in ERα positive breast cancer, RUNX1 phosphorylation involving in osteolytic bone destruction.

Recently, numerous studies have indicated that PAK4 involving in tumor metastasis. It has been shown that PAK4 phosphorylating Slug to promote EMT in prostate cancer 7. Furthermore, PAK4 promotes hepatocellular carcinoma metastasis via directly phosphorylating p53 on Ser215 49. It has been revealed that PAK4 phosphorylates SCG10 at Ser50 modulates gastric cancer metastasis 50. Studies have pointed out that PAK4 modulates ovarian cancer tumor progression via c-Src/ERK1/2 and EGFR/MMP2 signal pathway 51. Previous studies identified the nuclear PAK4 promotes bone metastasis in ERα positive breast cancer by targeting LIFR 10 and we demonstrated that MCF-7 cells overexpressed PAK4 promote metastatic bone colonization in mice, although MCF-7 was known as a low metastatic potential breast cancer cells. The low metastatic potential breast cells such as MCF7 and SUM159 all showed high-expression of LIFR, which involving in bone metastases 52,53, and both PAK4 and RUNX1 regulate the expression of LIFR^10^,^54^, suggest that PAK4 and RUNX1 may be involved in regulating the metastasis of breast cancer cells with low metastatic potential. As a kinase, PAK4 plays a biological role mainly by phosphorylating downstream substrates 55, but how PAK4 in ERα-positive breast cancer cells participates in osteolytic bone destruction in a kinase-dependent manner is still unclear and needs further verification. Here, we found that PAK4 promotes ERα-positive breast cancer-induced osteolytic bone destruction by phosphorylating RUNX1.

Overall, we discover a new mechanism for ERα-positive breast cancer-induced osteolytic bone destruction. Here, we demonstrate that PAK4 modulate the regulation of RUNX1 and that the nuclear PAK4 involving in ERα-positive breast cancer-induced osteolytic bone destruction. Our results provide that PAK4 could be a novel therapeutic target for ERα-positive breast cancer treatment.

## Supplementary Material

Supplementary figures and tables.

## Abbreviations

PAK4: P21 activated kinase 4
RUNX1: Runt-related transcription factor 1
PRMT1: Protein arginine methyltransferases 1
HDAC1: Histone Deacetylase 1
BMBC: Bone Metastatic Breast Cancer
