# Supplementary material for: PAK4 phosphorylating RUNX1 promotes ERα-positive breast cancer-induced osteolytic bone destruction
Source: Int J Biol Sci. 2020 May 25;16(12):2235–47. doi: 10.7150/ijbs.47225 (PMC7294946; doi:10.7150/ijbs.47225)

Figure 1. Phosphorylation of RUNX1 at T207 induces its translocation from the nucleus to the cytoplasm

(A) Endogenous PAK4 and RUNX1 were evaluated in ZR-75-30 cells. Cell lysates were immunoprecipitated with RUNX1 antibodies or IgG. Precipitates were analyzed by western blot using the indicated antibodies.

(B) Western blot of RUNX1 from the nuclear and cytoplasmic fractions obtained from human ZR-75-30 cells stably overexpression or knockdown of PAK4. β-tubulin and LaminB1 were used as controls for the cytoplasmic and nuclear compartments, respectively. Insets represent western blot analyses of PAK4

(C) Western blot of RUNX1 from the nuclear and cytoplasmic fractions obtained from human ZR-75-30 cells transfected with Flag-PAK4 WT, Flag-PAK4 NE, or Flag-PAK4 KM, β-tubulin and LaminB1 were used as controls for the cytoplasmic and nuclear compartments, respectively. Insets represent western blot analyses of the exogenous FLAG-tagged proteins.

(D) Western blot of RUNX1 from the nuclear and cytoplasmic fractions obtained from human ZR-75-30 cells transfected with Flag-RUNX1 T207D, Flag-RUNX1 WT or Flag-RUNX1 T207A, β-tubulin and LaminB1 were used as controls for the cytoplasmic and nuclear compartments, respectively. Insets represent western blot analyses of the exogenous FLAG-tagged proteins.

(E) MCF-7 cells were treated with increasing dose of E2, RUNX1 expression was tested by western blot.

Supplementary Figure 1

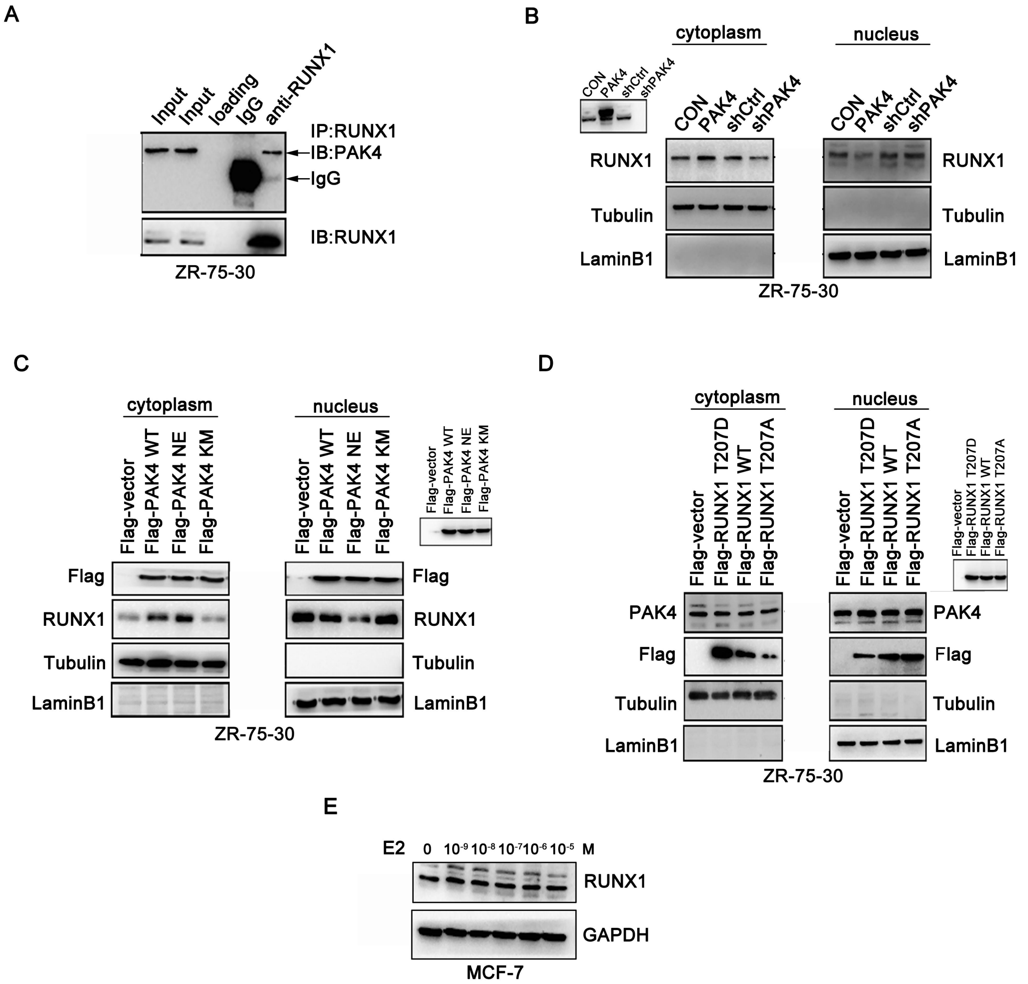

Figure 2. **Phosphorylation of RUNX1 at T207 influences interaction with both SIN3A/HDAC1 and PRMT1**

(A) Endogenous RUNX1, SIN3A, HDAC1 and PRMT1 were evaluated in ZR-75-30 cells which stably overexpressed PAK4. MCF-7 cell lysates were immunoprecipitated with the RUNX1 antibodies or IgG. Precipitates were analyzed by western blot using the indicated antibodies.

(B) ZR-75-30 cells transfected with Flag-PAK4 WT, Flag-PAK4 NE, or Flag-PAK4 KM, MCF-7 cell lysates were immunoprecipitated with the RUNX1 antibodies or IgG. Precipitates were analyzed by western blot using the indicated antibodies.

(C) ZR-75-30 cells transfected with Flag-RUNX1 WT, Flag-RUNX1 T207A or Flag-RUNX1 T207D. Cell lysates were immunoprecipitated with the Flag antibodies. Precipitates were analyzed by western blot using the indicated antibodies.

Supplementary Figure 2

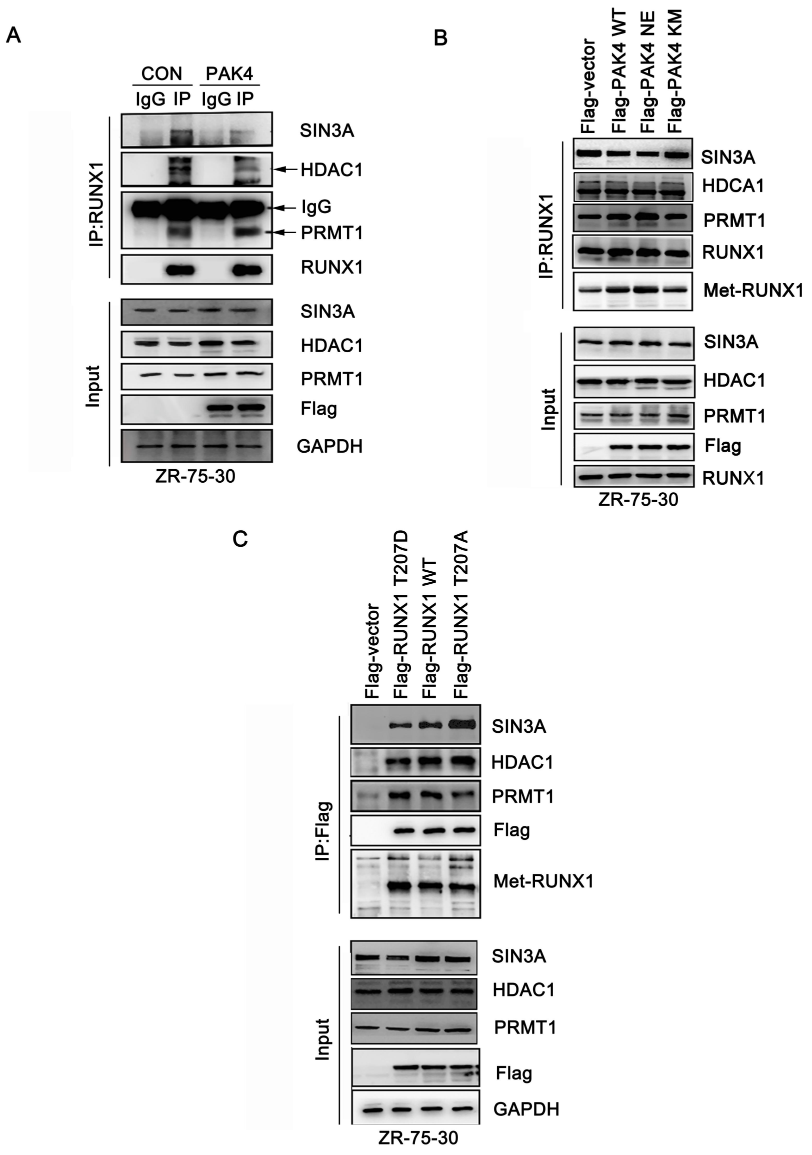

Supplement: Supplementary file 1 — Supplementary figures and tables. [file ijbsv16p2235s1.pdf]
